# A Polymeric Vesicle System for Combined Lung Cancer Therapy through Chemotherapy and Vasculature Normalization

**DOI:** 10.34133/bmr.0117

**Published:** 2024-11-27

**Authors:** Ding Wang, Cheng-Jie Qiu, Yaoqing Chu, Anzhuo Zhang, Ran Huang, Si-Jian Pan, Lianjiang Tan

**Affiliations:** ^1^ School of Materials Science and Engineering, Shanghai Institute of Technology, Shanghai 201418, China.; ^2^ Department of Neurosurgery, Ruijin Hospital, Shanghai Jiao Tong University School of Medicine, Shanghai 200025, China.; ^3^ Academy for Engineering and Technology; Yiwu Research Institute; Zhuhai Fudan Innovation Institute, Fudan University, Shanghai 200433, China.; ^4^ Center for Innovation and Entrepreneurship, Taizhou Institute of Zhejiang University, Taizhou, Zhejiang 318000, China.

## Abstract

Lung cancer remains a great threat to human health despite the rapid development of various therapeutic methods. Chemotherapy continues to be the most commonly employed treatment for lung cancer; however, it often suffers from low drug delivery efficiency and severe side effects. To enhance the therapeutic efficacy of chemotherapy, we developed a novel strategy that integrates tumor vasculature normalization with the co-delivery of therapeutic agents. This strategy employs a diblock polymeric vesicle with a reduction-sensitive linkage. Paclitaxel (PTX) is encapsulated in the bilayer, while an acid-sensitive nitric oxide (NO) precursor, DETA NONOate, and zinc oxide nanoparticles (ZnO NPs) are loaded into the central cavity. The resulting nanosystem, (ZnO,NONO)@Ves-PTX, is designed to release NO under the acidic conditions typical of the tumor microenvironment (TME) and intracellular environment. The released NO in the TME inhibits angiogenesis, thereby facilitating the delivery and distribution of therapeutic agents. Upon internalization by tumor cells, (ZnO,NONO)@Ves-PTX decomposes in response to intracellular glutathione (GSH), releasing the loaded agents. DETA NONOate and ZnO NPs generate NO and Zn^2+^ ions, respectively, at the intracellular pH, which synergistically inhibit tumor growth alongside PTX. This combined therapeutic approach demonstrated remarkable potential in improving the chemotherapeutic efficacy for lung cancer, offering a promising direction for future cancer treatments.

## Introduction

Lung cancer remains one of the leading causes of cancer-related mortality worldwide, primarily due to the limitations of current therapeutic strategies. Despite advances in various treatment modalities such as photothermal therapy [1], photodynamic therapy [2], immunotherapy [3], and their combinations [4–6], chemotherapy continues to be the cornerstone of lung cancer management [7–9]. However, traditional chemotherapy faces enormous challenges, including poor drug delivery efficiency, nonspecific distribution, and severe side effects, which collectively reduce therapeutic efficacy and patient quality of life. To overcome these challenges, researchers have increasingly turned to combination therapies and co-delivery systems that aim to enhance drug delivery and synergistically attack tumors through multiple mechanisms. Among these, nanocarrier-based systems have shown promise by improving the targeting and release of therapeutic agents at tumor sites [10]. However, these systems often face limitations in penetrating the dense and poorly perfused tumor microenvironment (TME) [11,12], which is characterized by abnormal vasculature and hypoxia [13,14].

In this context, tumor vasculature normalization has emerged as a crucial strategy to improve drug delivery within the TME [15–17]. Anti-angiogenic agents that remodel tumor vasculature and promote vessel maturation have been employed to modulate the TME [18–22]. Nitric oxide (NO), a versatile signaling molecule [23–25], has been identified as a key modulator of angiogenesis and tumor vasculature normalization [26–29]. By promoting the maturation of tumor vessels, NO can enhance blood perfusion and oxygenation, thereby improving the delivery and efficacy of chemotherapeutic agents [30,31].

Currently, chemotherapy using paclitaxel (PTX) and platinum-based drugs such as cisplatin and carboplatin is a prevalent clinical treatment method for lung cancer. However, these platinum drugs are associated with various adverse effects, including anaphylaxis, neurotoxicity, gastrointestinal reactions, nephrotoxicity, and hematotoxicity. Recent studies have demonstrated that zinc oxide nanoparticles (ZnO NPs) exhibit dose-dependent anticancer activity against lung cancer [32–34]. ZnO NPs induce membrane damage, increase oxidative stress, and decrease mitochondrial membrane potential in lung cancer cells. Additionally, ZnO NPs dissolve under the acidic conditions characteristic of the intratumoral and intracellular environment, releasing Zn^2+^ ions that are toxic to cancer cells [35]. In contrast, ZnO NPs and the released Zn^2+^ ions cause minimal damage and toxicity to normal cells. Thus, ZnO NPs may represent a promising alternative to platinum drugs in lung cancer chemotherapy.

**Fig. 1.**
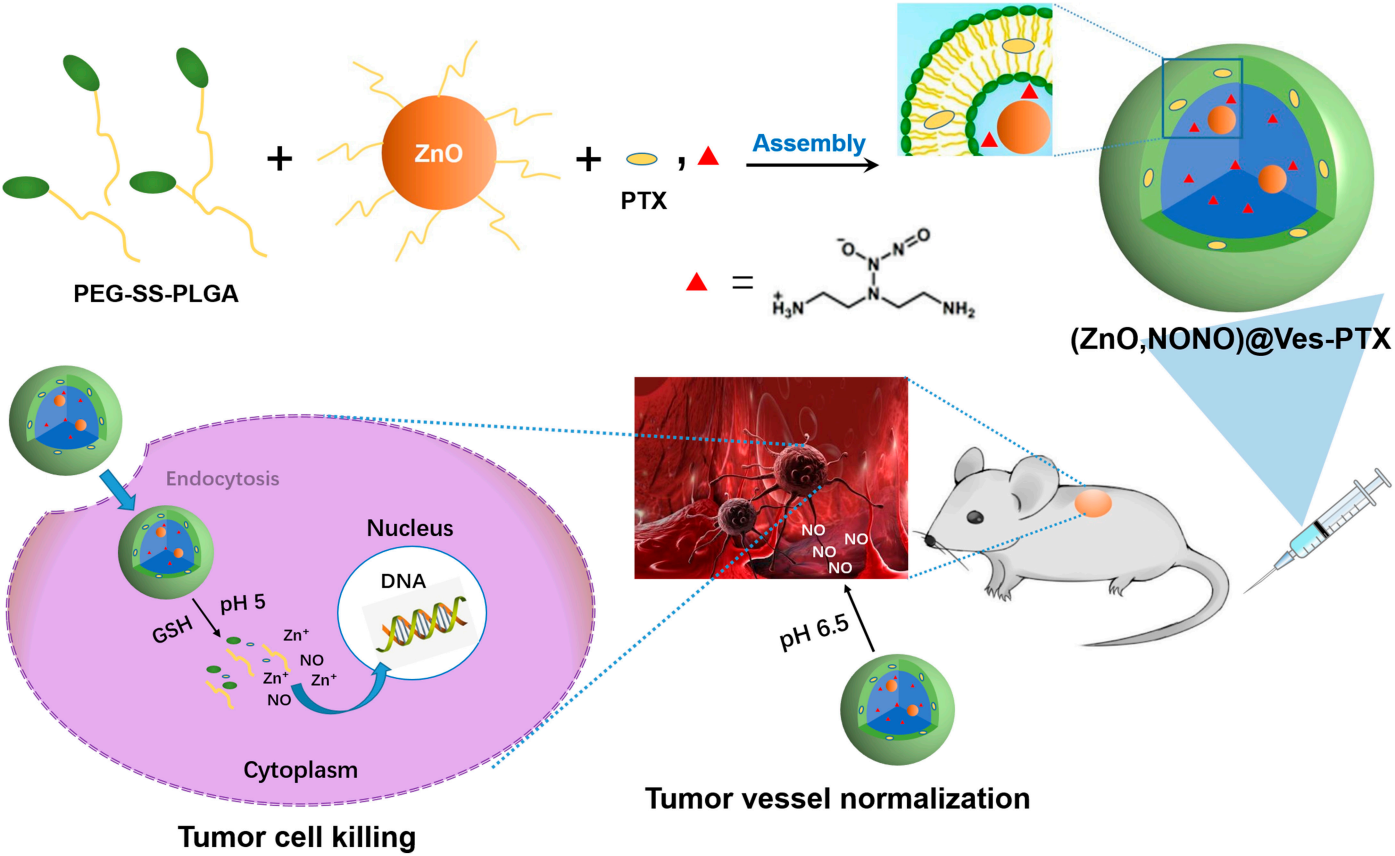
Schematic illustration of combining tumor vessel normalization and synergistic antitumor therapy using a decomposable block polymer vesicle.

In this study, we developed a diblock polymeric vesicle system, denoted as (ZnO,NONO)@Ves-PTX, designed to co-deliver NO, ZnO NPs, and PTX in a controlled manner. This system leverages the acidic conditions of the TME and intracellular glutathione (GSH) levels to trigger the release of these therapeutic agents, thereby enhancing the antitumor effects through both chemotherapy and vasculature normalization. As depicted in Fig. 1, the polyethylene glycol (PEG)-capped ZnO NPs and DETA NONOate, both hydrophilic, were encapsulated in the central cavity of the vesicles. The hydrophobic PTX was incorporated into the bilayer of the vesicles. The composite vesicle [(ZnO,NONO)@Ves-PTX] remained stable in TME, with the encapsulated DETA NONOate partially decomposing at lowered pH to release NO. Upon internalization by tumor cells, the vesicles disassembled in response to high concentrations of GSH, releasing ZnO NPs and PTX. The dose of NO released in the TME was sufficient to normalize tumor vessels, creating a more favorable environment for drug delivery. Inside the tumor cells, the ZnO NPs dissolved, releasing zinc ions that, along with PTX, destroyed the tumor cells. Additionally, the intracellular acidic conditions facilitated the decomposition of more DETA NONOate molecules, releasing a larger dose of NO than in the extracellular TME, thereby synergistically killing the tumor cells. This study provides a comprehensive evaluation of the (ZnO,NONO)@Ves-PTX system through in vitro and in vivo experiments, demonstrating its potential to substantially improve lung cancer treatment outcomes. By addressing the dual challenges of drug delivery and tumor resistance, our approach offers a promising avenue for future cancer therapies.

## Materials and Methods

### Materials

Zinc acetate (ZnAc_2_·2H_2_O) and PEG_400_ were supplied by Sinopharm Chemical Reagent Co. Ltd. PTX (99%) was purchased from Beijing Huafeng United Technology Corporation. PEG_2000_-SS-PLGA_1000_ was provided by SUNR Bio-Nanotechnology Co. Ltd. according to our requirement. DETA NONOate was provided by MedChemExpress. Calcein AM and doxorubicin hydrochloride were purchased from Aladdin Co. Ltd. Dimethyl sulfoxide (DMSO) and dimethyl formamide (DMF) were supplied by Adamas Co. Ltd. VEGF165 angiogenesis cytokine, Anti-angiogenin 2, Anti-VEGF was purchased from Abcam. Simulated body fluid (SBF; pH 7.4) containing 50 mM trishydroxymethy laminomethane and 45 mM hydrochloric acid, Dulbecco’s modified Eagle’s medium high glucose (DMEM), and 3-(4,5-dimethylthiazol-2-yl)-2,5-diphenyl-2*H*-tetrazolium bromide (MTT) were supplied by Sigma-Aldrich Co. Ltd. Other solvents used were provided by Sinopharm Chemical Reagent Co. Ltd. Deionized water was used during the experiment from Millipore water purification system (Merck, Plus185).

### Synthesis of ZnO NPs

Typically, 5 mmol of ZnAc_2_·2H_2_O was dissolved in 80 ml of anhydrous ethanol, stirred for 3 h at 60 °C. Meanwhile, 6 mmol of NaOH was dissolved in 25 ml of ethanol at 60 °C, which was added into the above mixture under stirring, refluxed at 60 °C for 1 h. Then the resultant solution was kept at 0 °C for 10 h to allow the growth of ZnO nanocrystals. ZnO NPs were precipitated by addition of N-hexane, which were then mixed with appropriate amounts of PEG_400_ under stirring for 3 h. Thereafter, the solution was centrifuged at 12,000 rpm for 5 min, and PEG-capped ZnO NPs were obtained.

### Preparation of (ZnO,NONO)@Ves-PTX

The amphiphilic copolymer PEG_2000_-SS-PLGA_1000_ and PTX were dissolved in chloroform at 25 °C. Under nitrogen atmosphere, the chloroform was evaporated at 60 °C, and vesicles with PTX loaded were formed. After thoroughly removing the chloroform, the vesicles were resuspended in a 0.1 M ammonium sulfate aqueous solution under magnetic stirring for 1 h. Then, the vesicles were dialyzed against a phosphate-buffered saline (PBS) buffer (pH 7.4) for 8 h. Thereafter, the aqueous solution containing PEG-capped ZnO NPs and DETA NONOate was added, stirred for 1 h. Finally, the solution was dialyzed against the PBS buffer for 5 h, and (ZnO,NONO)@Ves-PTX was obtained. The parallel samples of Ves-PTX, NONO)@Ves, (ZnO,NONO)@Ves, ZnO@Ves-PTX, and NONO@Ves-PTX, which were used for comparison, were prepared with the method described above, except for the different formulations.

Since the parallel samples were prepared as per the same protocols as (ZnO,NONO)@Ves-PTX, the drug loading consistency was ensured. The loading content of ZnO NPs in (ZnO,NONO)@Ves-PTX and the parallel samples was 5.4 ± 0.4 wt %, determined by thermogravimetric analysis (TGA). The loading contents of PTX and DETA NONOate were 8.7 ± 0.5 wt % and 5.9 ± 0.5 wt %, respectively, determined by ultraviolet–visible (UV–vis) spectroscopy after (ZnO,NONO)@Ves-PTX had been broken by sonication.

### Characterization

A JEM-2100 transmission electron microscope (TEM; JEOL, Japan) was used to observe the sample morphology. Samples were dispersed in absolute ethanol and dropped on a carbon-coated copper grid, which was then freeze dried in vacuum at −50 °C before observation. X-ray diffraction (XRD) pattern was recorded by a D/max-2200/PC X-ray diffractometer (Rigaku, Japan). Hydrodynamic size (*Z*-average diameter), size distribution, and zeta potential of the samples were measured by a Nano ZS90 particle size and zeta potential analyzer (Malvern, UK) based on dynamic light scattering (DLS). ^1^H nuclear magnetic resonance spectroscopy (^1^H NMR) was performed on a Mercury Plus-400 spectrometer at 400 MHz (Varian, USA).

### Measurement of PTX release

The amount of PTX released from (ZnO,NONO)@Ves-PTX was determined by high-performance liquid chromatography (HPLC). (ZnO,NONO)@Ves-PTX was freeze dried and then dissolved in PBS buffers containing GSH of different concentrations (pH 7.4, 37 °C). The resultant solutions were transferred into a dialysis tube [molecular weight cutoff (MWCO) = 500 Da]. The release of PTX was measured according to the method reported in our previous work [36].

### Measurement of Zn^2+^ ion release

A dialysis tube (MWCO = 500 Da) containing 10 ml of 100 μg/ml (ZnO,NONO)@Ves-PTX was immersed in 100 ml of PBS buffer at different pH values at 37 °C. The solution (5 ml) outside the dialysis tube was sampled at predetermined time points. The Zn^2+^ ions in the samples were quantified using an inductively coupled plasma mass spectrometer (ICP-MS) (Thermo Elemental, UK).

### Measurement of NO release

The amount of NO released from (ZnO,NONO)@Ves-PTX at varied pH values was measured by a TBR 4100/1025 free radical analyzer equipped with an ISO-NOP sensor (WPI Ltd., USA). The NO detection was conducted as per the protocols described in our previous work [37].

### Cell culture

Human pulmonary carcinoma A549 cells, human umbilical vein endothelial cells (HUVECs), and mouse embryo fibroblast NIH 3T3 cells were provided by the Institute of Biochemistry and Cell Biology, Chinese Academy of Science. The cells were cultured in Dulbecco’s modified Eagle’s medium (DMEM) supplemented with 10 wt % fetal bovine serum, 100 IU/ml penicillin, and 100 μg/ml streptomycin in a humidified incubator with 5 vol % carbon dioxide at 37 °C. The medium was refreshed every other day as per cell density.

### MTT assay

HUVECs or A549 cells were seeded in 96-well culture plates at a density of ~5,500 cells/well, incubated for 24 h at 37 °C. Then, the culture medium was replaced by a fresh medium containing different drug formulations. After incubation for an additional 24 h, the cells were treated with 150 μl of MTT (100 μg/ml) for 4 h. Afterward, the medium was removed and 150 μl of DMSO was added. After incubation for 10 min, the absorbance of the mixture at 570 nm was measured using a plate reader (AF2200, Germany). The cell viability was thus determined.

### Vascular endothelial growth factor expression assay

Cultured in a VEGF-MV Complete Kit, the HUVECs were incubated with different formulations at an equivalent vesicle concentration of 100 μg/ml for 24 h. Untreated cells were taken as a control. Before incubation, the pH was adjusted to 6.5. Then, the cells were centrifuged to collect the supernatant. The vascular endothelial growth factor (VEGF) expression was obtained by the use of an enzyme-linked immunosorbent assay (ELISA) kit (Merck, Germany).

### Western blot analysis

The cultured HUVECs were treated with different formulations (pH 6.5) at the equivalent vesicle concentration of 100 μg/ml for 12 h. Then, the cells were washed and lysed in ice-cold radioimmunoprecipitation assay buffer containing protease and phosphatase inhibitors. The cell lysates were loaded onto 12% sodium dodecyl sulfate–polyacrylamide gel electrophoresis (SDS-PAGE) gel, electro-transferred to polyvinylidene fluoride (PVDF) membranes, and were processed following the protocols reported in our previous work [38]. The antibody dilutions used for the Western blot studies were as follows: anti-VEGF (1:1,000), anti-basic fibroblast growth factor (bFGF) (1:1,000), anti-CD31 (1:1,000), and β-actin (1:5,000).

### Wound healing assays

In the wells of 6-well plates, HUVECs were seeded at a density of ~10^6^ cells/well. A sterile pipette tip was used to scratch the cell layer, and the cells were washed with PBS to remove unattached ones. Afterward, the cells were treated with 120 μg/ml (ZnO,NONO)@Ves-PTX for 24 h after washing with serum-free medium 3 times. Then the migration of the cells was monitored on an inverted microscope (Ti-U, Nikon). The culture plates were photographed at 6 and 12 h. The migration rate of the HUVECs was calculated based on the control group.

### Tube formation assay

Different formulations were added to the 24-well plates where HUVECs had been incubated overnight at a density of ~10^4^ cells. The pH in each well was adjusted to 6.5. Incubated for 24 h, the HUVECs were seeded on 10 μl Matrigel-coated (Corning) μ-Slide Angiogenesis (ibidi). In order to promote the tube formation, 40 ng/ml VEGF was added to all the groups except the control group. Then, 6 μM calcein AM stock solution was added to each well, and the cells were cultured at 37 °C and 90% humidity in 5% CO_2_ for 20 min. The angiopoiesis process was observed by the inverted microscope (Ti-U, Nikon) under 488-nm excitation. After 24 h, fluorescence images were acquired and analyzed using ImageJ*.* The tube formation parameters were determined by the Angiogenesis Analyzer.

### In vitro cell assay

A549 cells were seeded in 6-well plates at a density of 6 × 10^5^ cells/well in 2 ml of complete DMEM and cultured for 24 h. The cells were washed by a PBS buffer (pH 7.4) and then incubated with (ZnO,NONO)@Ves-PTX in the complete DMEM (10 μg/ml) for 2 or 10 h at 37 °C. Then, some of the cells were transferred to an 8-well Lab-Tek II chamber slide (Nalge Nunc, Napevillem, IL). The medium was aspirated from the wells, and the cells were rinsed with the PBS buffer for 3 times. The cell fluorescence was observed by a confocal laser scanning microscope (Zeiss LSM 710, Germany). The remaining cells in the 6-well plates were rinsed with the PBS buffer for 3 times and treated with trypsin. The resultant cell suspension was centrifuged at 2,000 rpm for 5 min. After removal of supernatants, the cells were resuspended in 2 ml of the PBS buffer and observed by a flow cytometry (BD FACSCalibur, USA).

### Animal tumor modeling

Eight-week-old female BALB/c mice with a body weight of ~22 g, which were provided by Shanghai Institute of Zoology, Chinese Academy of Science, were used to build a mouse xenograft model of A549 tumor. All the in vivo experiments were conducted as per protocols approved by the Animal Ethics Committee of Shanghai Jiao Tong University (protocol approval number 201902019). The mice were divided into 7 groups randomly. A549 cells were injected subcutaneously in the right flank region of the mice. When the inoculated A549 tumors grew to a volume of ~150 mm^3^, the mice were treated with tail vein injection of PBS (200 μl, control) and different formulations (200 μl, 20 mg vesicle/kg) over a period of 21 d in total. The tumor volumes and body weights of each group were measured every 3 d. On the one hand, the harvested tumors on the 21st day were dissected and the tumor tissue was cleared using a clearing solution, incubated at 37 °C for 48 h. Then, the tumor samples were gently removed from the clearing solution and fixed on a microscope glass slide. A small drop of clearing solution was put on the samples, and then a glass coverslip was placed on top of the samples, with gentle pressure applied. The inverted microscope was used to observe the vasculature network of the tumors.

On the other hand, the tissues of harvested tumors were embedded in paraffin, sectioned, and stained with hematoxylin and eosin (H&E) for histopathology analysis. The survival rate of the mice with different treatments was determined by monitoring the vital signs of the mice over a period of 50 d.

The distribution of (ZnO,NONO)@Ves-PTX in the mouse body was investigated. At predetermined time points, the mice were sacrificed, whose organs and tumor were harvested and treated with hydrochloric acid. The resultant samples were dried and decomposed in hot nitric acid and perchloric acid. The Zn concentration in the samples was determined by ICP-MS. In addition, a liver and a spleen were fixed in a 4% paraformaldehyde (PFA) solution. The resultant tissues were embedded in paraffin, sectioned, and stained with H&E for histological analysis.

### Blood analysis

The mice were anesthetized, whose tails were wiped with warm water. The tail tip (2 to 3 mm) was cut off, and the whole blood was collected in centrifuge tubes with heparin in them. The whole blood samples were analyzed by a Chemray 800 biochemical analyzer (Rayto, USA).

### Statistical analysis

All data were represented as mean ± SD. Statistical significance was analyzed using Student’s *t* test. The data were considered as statistically significant at *P < 0.05 or **P < 0.01. When more than 2 groups were compared, a one-way analysis of variance (ANOVA) followed by Bonferroni’s post hoc test was used for significance evaluation.

## Results

### Preparation and characterization of (ZnO,NONO)@Ves-PTX

**Fig. 2.**
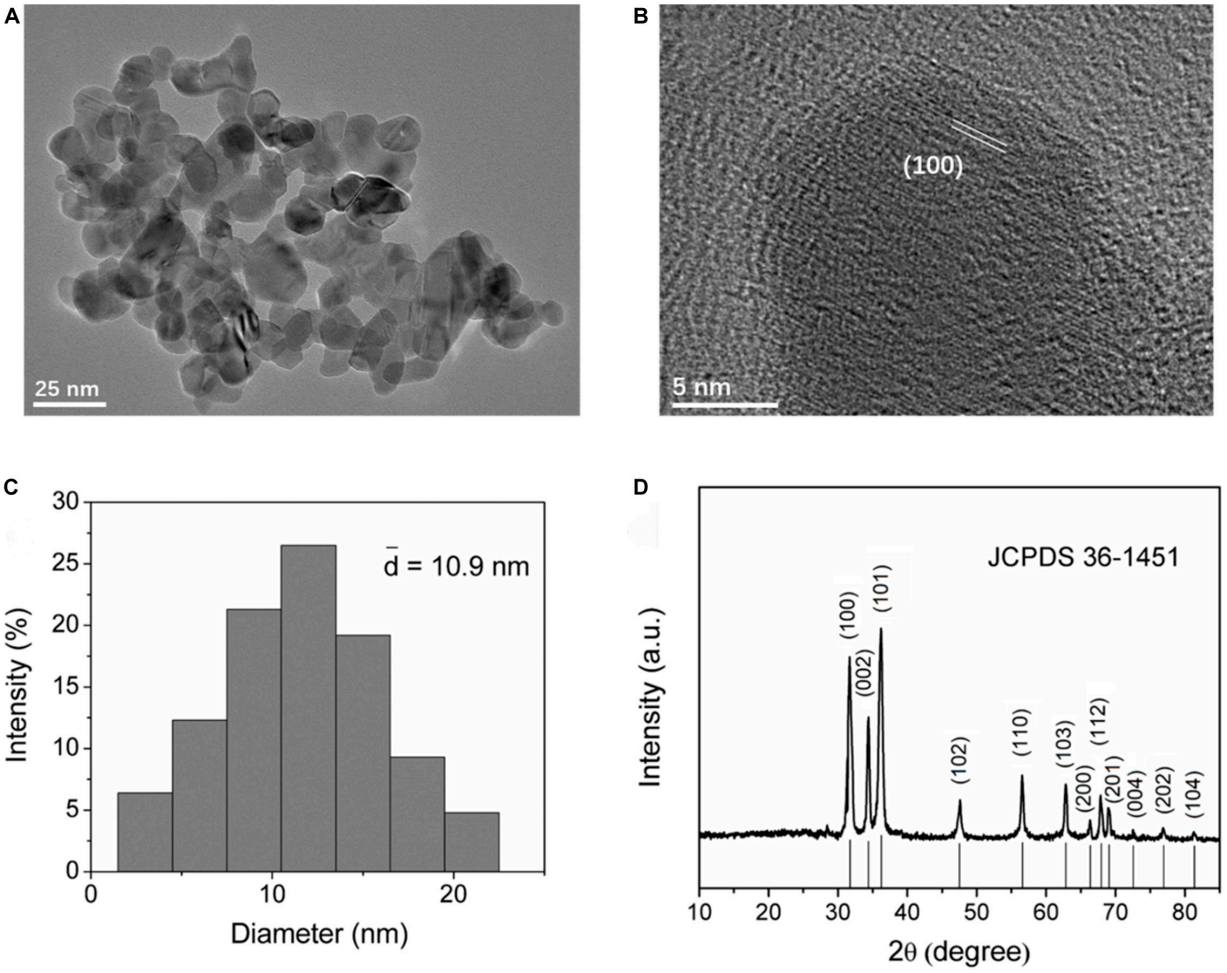
(A) TEM image, (B) high-resolution TEM image, (C) size distribution histograms, and (D) XRD pattern of ZnO NPs.

ZnO NPs were synthesized via a wet chemical method and were observed by TEM. The images of the resultant ZnO NPs are shown in Fig. 2A and B. The ZnO NPs were nanospheres with uniform size (Fig. 2A). The TEM image at a larger magnification (Fig. 2B) displays the morphology of the ZnO NPs, from which the lattice fringes of (100) crystal planes can be clearly seen. We measured the size of at least 50 particles in a randomly selected zone of a TEM image, based on which the size distribution histograms of the ZnO NPs were obtained. As shown in Fig. 2C, the ZnO NPs had an average diameter of 10.9 nm. The XRD pattern in Fig. 2D showed main diffraction peaks indexed, which were in accordance with those of the wurtzite structure of ZnO (JCPDS Card No. 36-1451).

**Fig. 3.**
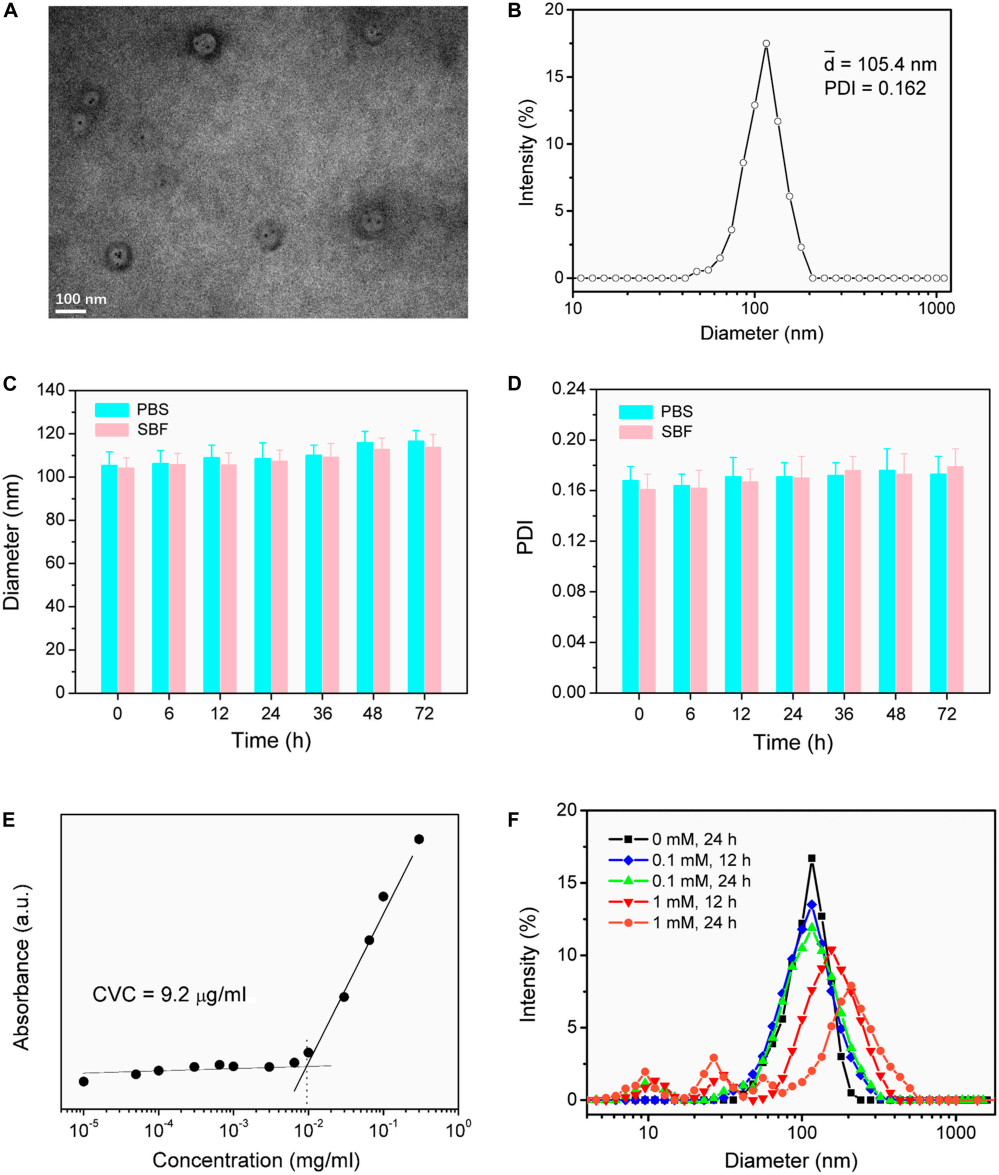
(A) TEM image and (B) DLS size distribution of (ZnO,NONO)@Ves-PTX. (C) Diameter and (D) PDI variations of (ZnO,NONO)@Ves-PTX in PBS and SBF, respectively, over a period of 72 h. The data are presented as average ± SD (*n* = 4). (E) Plot of absorbance of DPH versus concentration of (ZnO,NONO)@Ves-PTX in water. CVC was determined by the crossover of the 2 fitting lines. (F) Size changes of (ZnO,NONO)@Ves-PTX in a PBS buffer (pH 7.4, 37 °C) at varied GSH concentrations over a period of 12 and 24 h.

The amphiphilic copolymer PEG_2000_-SS-PLGA_1000_, whose ^1^HNMR is shown in Fig. S1, was used to load multiple therapeutic agents. The copolymer self-assembled into vesicles, encapsulating the agents either in the bilayer or in the central cavity. The prepared (ZnO,NONO)@Ves-PTX was observed by TEM. As shown in Fig. 3A, typical morphology of vesicles reveals the structure of (ZnO,NONO)@Ves-PTX. The dots in the central cavity of the vesicles were ZnO NPs. The hydrodynamic size distribution of (ZnO,NONO)@Ves-PTX in deionized water was determined by DLS. As shown in Fig. 3B, the average hydrodynamic diameter was 105.4 nm, and the polydispersity index (PDI) was 0.162. The zeta potential was determined on the same instrument, the results of which are shown in Fig. S2. (ZnO,NONO)@Ves-PTX was negatively charged. The average zeta potential of −26.2 mV suggested the dispersion stability of the nanoscaled vesicles.

At pH 7.4 and 37 °C, PBS and SBF are good candidates for simulating physiological environment. We monitored the hydrodynamic particle size and PDI of (ZnO,NONO)@Ves-PTX in the 2 solutions for 72 h so as to evaluate its long-term colloidal stability. As indicated in Fig. 3C and D, the diameter of (ZnO,NONO)@Ves-PTX in both solutions changed only slightly during the period of 72 h. PDI also showed little variation in the 2 media. Moreover, the critical vesicle concentration (CVC) of (ZnO,NONO)@Ves-PTX, which is a measure of vesicle stability, was determined using a hydrophobic UV probe, 1,6-diphenyl-1,3,5-hexatriene (DPH). As shown in Fig. 3E, the changes of UV absorbance with the vesicle concentration could be divided into 2 parts, the fitting lines of which crossed over at a point. The corresponding vesicle concentration was regarded as CVC, which turned out to be 9.2 μg/ml. That means, (ZnO,NONO)@Ves-PTX keeps vesicle structure at concentrations higher than 9.2 μg/ml.

To investigate the responsiveness to GSH, we measured the hydrodynamic size changes of (ZnO,NONO)@Ves-PTX with time at different GSH concentrations (pH 7.4, 37 °C), as shown in Fig. 3F. Without GSH in the PBS buffer, the average diameter hardly changed after incubation for 24 h, indicating the stability of (ZnO,NONO)@Ves-PTX under physiological conditions. In the presence of 1 mM GSH, the main peak of the size distribution curve changed slightly with incubation time. However, a small peak centered at around 10 nm appeared at 24 h. This was most likely due to the decomposition of (ZnO,NONO)@Ves-PTX when the disulfide bonds broke in response to the GSH. When the GSH concentration was enhanced to 10 mM, 3 peaks at 11, 30, and 175 nm were observed after incubation for 12 h. The decomposition of (ZnO,NONO)@Ves-PTX resulted in molecules with smaller molecular weights and release of the encapsulated agents. The size distribution thus increased, leading to multiple peaks at different sizes. At 24 h, the size distribution became even wider, with 4 peaks on the size distribution curve. These results demonstrated the GSH-responsive decomposition of (ZnO,NONO)@Ves-PTX.

### Release of therapeutic agents

**Fig. 4.**
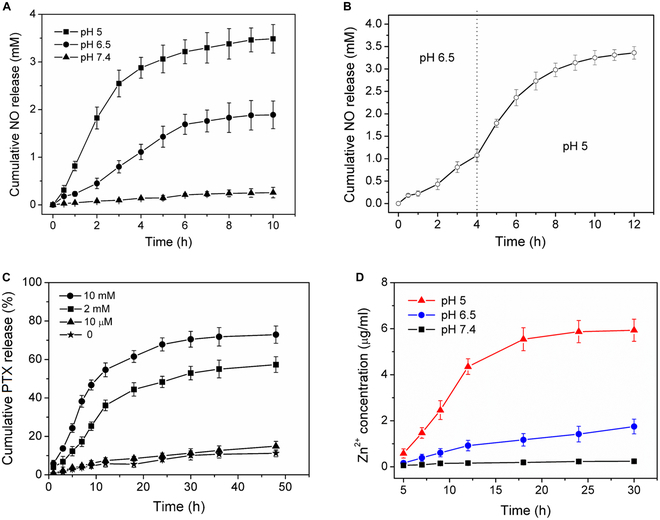
(A) Profiles NO release from (ZnO,NONO)@Ves-PTX in PBS buffers of different pH values at 37 °C. (B) Two stages of NO release from (ZnO,NONO)@Ves-PTX: pH 6.5 for the first 4 h and pH 5 for the subsequent 8 h. (C) PTX release profiles of (ZnO,NONO)@Ves-PTX in PBS buffer (pH 7.4, 37 °C) containing varied concentrations of GSH. (D) Zn^2+^ ions released from (ZnO,NONO)@Ves-PTX in PBS buffers of varied pH values at 37 °C. The GSH concentration in the PBS buffers was 10 mM. All data are presented as average ± SD (*n* = 4).

The hydrophilic NO donor DETA NONOate was encapsulated in the central cavity of the vesicles, endowing the (ZnO,NONO)@Ves-PTX pH-sensitive NO-release properties. There is actually a pH gradient inside the tumor. In extracellular TME, the pH is usually at around 6.5. While the intracellular pH can be as low as 5. To investigate NO-release behavior of (ZnO,NONO)@Ves-PTX at different pH values, the release profiles of NO are plotted. As shown in Fig. 4A, a very small amount of NO was released at the physiological pH 7.4. When the pH was lowered to 6.5, more NO was released, achieving a cumulative dose of 1.89 mM at 10 h. At pH 5 typical of intracellular endosomal and lysosomal systems, substantial NO release was detected, and the cumulative NO-release amount at 10 h was 3.48 mM. To simulate the NO release under intratumoral conditions, we further measured the NO release at pH 6.5 and pH 5, successively. As indicated in Fig. 4B, the NO release showed a 2-stage profile. The released amount of NO in the second stage was much larger than that in the first stage, revealing substantially different NO release between extra- and intracellular environment.

The PTX trapped in the bilayer of the vesicles will be released along with the decomposition of the vesicles. The GSH-responsive PTX release from (ZnO,NONO)@Ves-PTX is shown in Fig. 4C. When the GSH concentration was 10 μM that is typical of extracellular environment, little PTX was released, similar to the case without GSH. When the GSH concentration increased to 2 and 10 mM, a cumulative release of 57.3% and 72.9% NO was found at 72 h, respectively. The PTX release behavior indicated that (ZnO,NONO)@Ves-PTX is stable outside the tumor cells. Internalized by the tumor cells, however, the disulfide bonds are cleaved by the intracellular GSH of high concentrations (typically 2 to 10 mM in tumor cells). (ZnO,NONO)@Ves-PTX thus decomposes and releases PTX.

In acidic PBS buffers, the ZnO NPs in (ZnO,NONO)@Ves-PTX will dissolve and release Zn^2+^ ions. The concentration of released Zn^2+^ ions can be measured by ICP-MS. As shown in Fig. 4D, few Zn^2+^ ions were released at pH 7.4, as the ZnO NPs kept stable in the neutral environment. At pH 6.5, ZnO NPs were dissolved to some extent, releasing a small amount of Zn^2+^ ions. In the case of pH 5, more Zn^2+^ ions were generated, and the Zn^2+^ ion concentration was 5.93 μg/ml at 30 h. The pH-dependent release of Zn^2+^ ions suggested that most of the Zn^2+^ ions were released inside the tumor cells, which could act directly on the tumor cells synergistically with NO and PTX.

### Vasculature normalization of (ZnO,NONO)@Ves-PTX

**Fig. 5.**
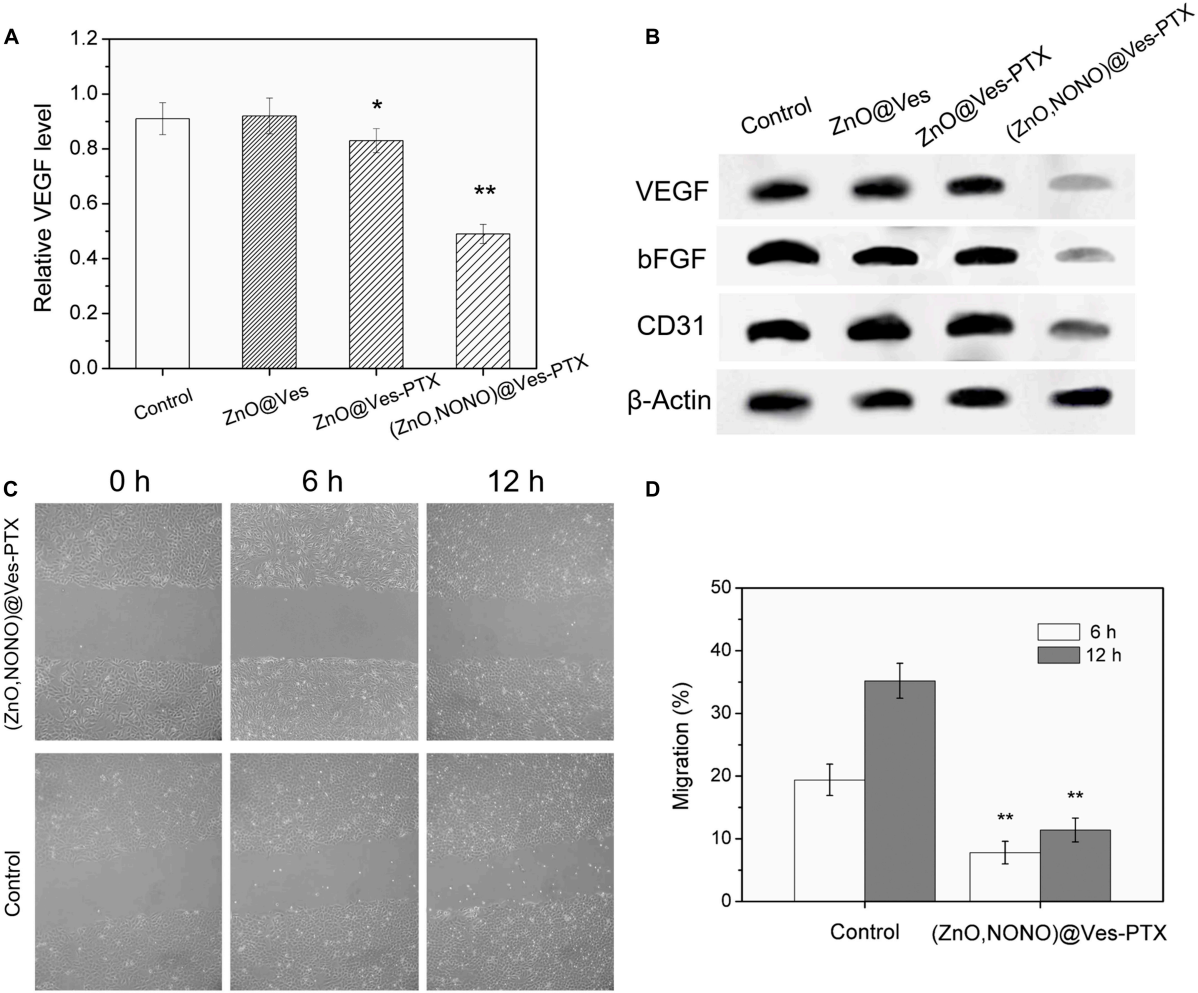
(A) VEGF inhibition of HUVECs by ZnO@Ves, ZnO@Ves-PTX, and (ZnO,NONO)@Ves-PTX (equivalent vesicle concentration of 100 μg/ml) measured using the ELISA kit at 24 h. (B) Expression levels of VEGF, bFGF, and CD31 in HUVECs treated with different formulations (equivalent vesicle concentration of 100 μg/ml), analyzed by Western blot. Untreated HUVECs were used as a control, and β-actin was the loading control. (C) Wound healing assay indicating the migration ability of HUVECs using Vasculife-MV complete kit (0, 6, and 12 h after treatment with PBS or (ZnO,NONO)@Ves-PTX (100 μg/ml vesicle). Scale bar, 200 μm. (D) Migration of HUVECs after treatment for 6 and 12 h. All the data are presented as average ± SD (*n* = 5). Statistical significance: *P < 0.05; **P < 0.01.

VEGF, an important angiogenesis factor, is closely related to tumor vessels. High levels of VEGF in tumors usually indicate vessel abnormalities [39]. To evaluate the vasculature normalization function of (ZnO,NONO)@Ves-PTX, cultured HUVECs were incubated with (ZnO,NONO)@Ves-PTX and other formulations for 24 h at pH 6.5. The VEGF expression levels of the HUVECs with different treatments are shown in Fig. 5A. Compared with the untreated control group, the group treated with ZnO@Ves and ZnO@Ves-PTX showed similar and a little lower VEGF expression, respectively. In contrast, the VEGF expression level was significantly lower when the HUVECs were treated with (ZnO,NONO)@Ves-PTX, with a decrease of 46.1% compared with the control group. At pH 6.5 typical of TME, some of the DETA NONOate decomposed to release NO, which decreased the VEGF expression of HUVECs.

bFGF and a marker of vascular endothelial cells, CD31, also have close relationships to tumor angiogenesis [40,41]. Western blotting was conducted to examine the effects of different formulations on the expression of VEGF, bFGF, and CD31. β-Actin was used as a loading control. Compared with the untreated HUVECs (Fig. 5B), the expression of bFGF, VEGF, and CD31 of the HUVECs was suppressed by (ZnO,NONO)@Ves-PTX. The expression of the 3 factors was not affected by the formulations without NO loaded. It is evident that the released NO from (ZnO,NONO)@Ves-PTX reduced the expression of the 3 factors.

Wound healing experiments were carried out to examine the effects of (ZnO,NONO)@Ves-PTX on cell migration at pH 6.5. Compared with the control group, the migration of HUVECs to the scratch zone was remarkably suppressed by (ZnO,NONO)@Ves-PTX, as shown in Fig. 5C. The migration rate of the cells, calculated based on the wound healing results, was reduced by 59.8% and 67.6% at 6 and 12 h, respectively, as revealed in Fig. 5D. The wound healing experiments demonstrated the ability of (ZnO,NONO)@Ves-PTX to suppress migration of HUVECs under weakly acidic conditions.

**Fig. 6.**
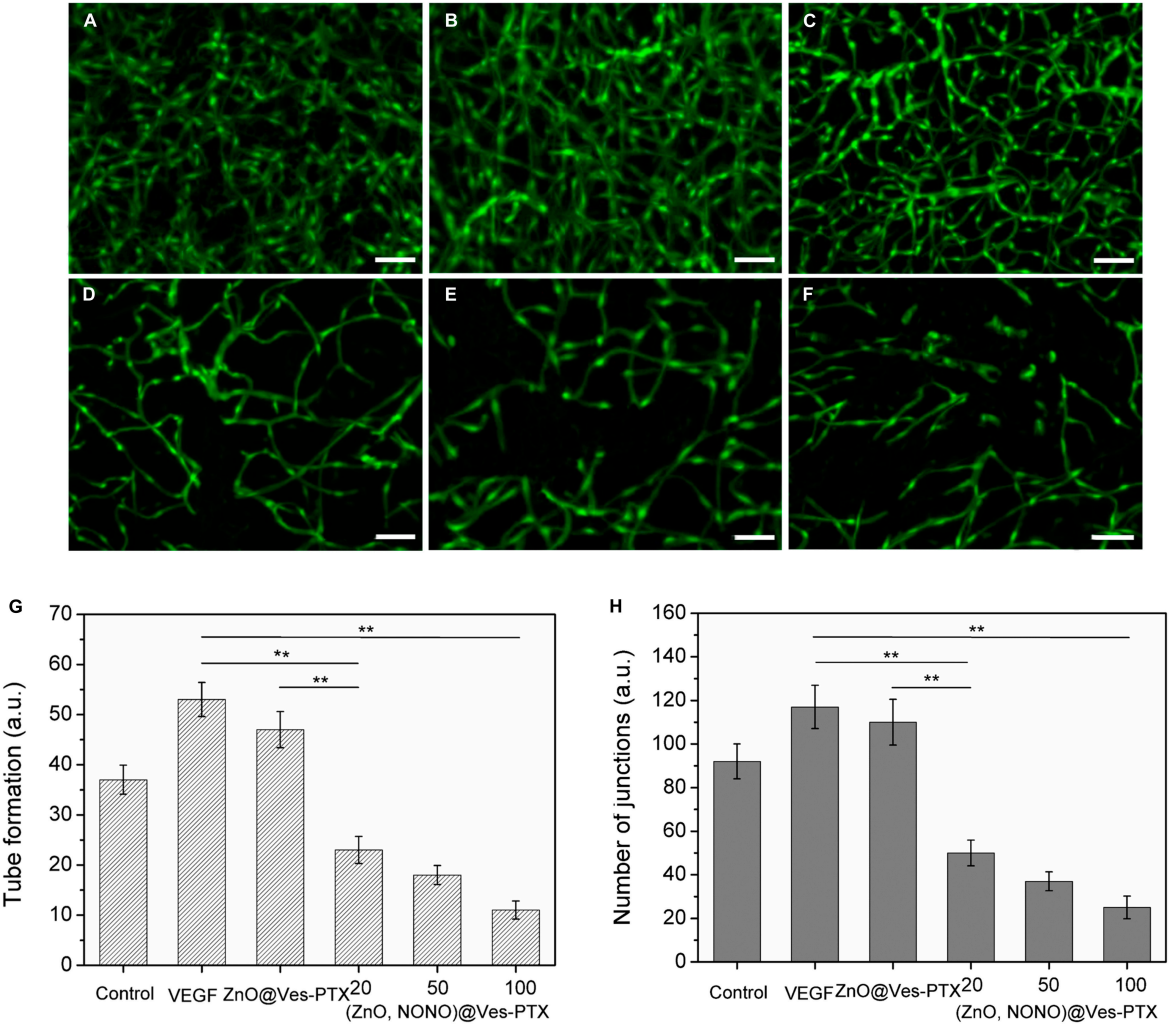
Fluorescence images of tube formation from HUVECs: (A) blank control, (B) VEGF, (C) VEGF + ZnO@Ves-PTX, (D) VEGF + (ZnO,NONO)@Ves-PTX (20 μg/ml vesicle), (E) VEGF + (ZnO,NONO)@Ves-PTX (50 μg/ml vesicle), and (F) VEGF + (ZnO,NONO)@Ves-PTX (100 μg/ml vesicle). Scale bar, 200 μm. Quantitative analysis of tube formation (G) and junctions (H) after different treatments by ImageJ based on the fluorescence images. The untreated blank group was taken as a control. The data are presented as average ± SD (*n* = 6). Statistical significance: **P < 0.01.

The vessel formation ability of HUVECs under the effects of ZnO@Ves-PTX and (ZnO,NONO)@Ves-PTX was investigated. The dynamic angiopoiesis process of calcein AM-stained HUVECs was observed by fluorescence microscopy, and fluorescence images were taken at the time point of 24 h. Treating HUVECs with VEGF was aimed at promoting vessel formation. As shown in Fig. 6A to F, the HUVECs treated with different formulations demonstrate vessel formation of different degrees. The number of formed tubes and junctions was analyzed by ImageJ, which are shown in Fig. 6G and H. The group treated with VEGF + ZnO@Ves-PTX showed slightly fewer vessels compared with the VEGF group. For the groups treated with VEGF + (ZnO,NONO)@Ves-PTX, the amount of vessels formed decreased dramatically. With the increase of concentration, the vessels were reduced to a larger extent. Quantitative analysis indicated that the number of tubes was 53 ± 3.4 and 47 ± 3.6 for the VEGF group and the group treated with ZnO@Ves-PTX, respectively. For the groups treated with (ZnO,NONO)@Ves-PTX, the number of tubes was 23 ± 2.7, 18 ± 1.9, and 11 ± 1.8 at the concentration of 20, 50, and 100 μg/ml, respectively. The number of junctions of the groups treated with (ZnO,NONO)@Ves-PTX was 50, 37, and 25 at the 3 concentrations, respectively (Fig. 6H), much smaller than that of the VEGF group. The tube formation results indicated that (ZnO,NONO)@Ves-PTX inhibited the tube formation of HUVECs, and the inhibitory effect originated from the NO.

Furthermore, the NO dose dependence of vasculature normalization was investigated by examining the changes in tube formation of HUVECs with NO concentration. The PEG_2000_-SS-PLGA_1000_ vesicles loaded with DETA NONOate were added to HUVECs, which had been treated with VEGF and were cultured at 37 °C and pH 6.5. The NO concentration in the cell suspension was measured, and the vessel formation was analyzed. As shown in Fig. S3, the tube formation of HUVECs was greatly reduced when the NO concentration increased from 0 to 20 μM. With the further increase of NO dose, the decrease of tube formation slowed down.

### Tumor cell uptake and inhibition

**Fig. 7.**
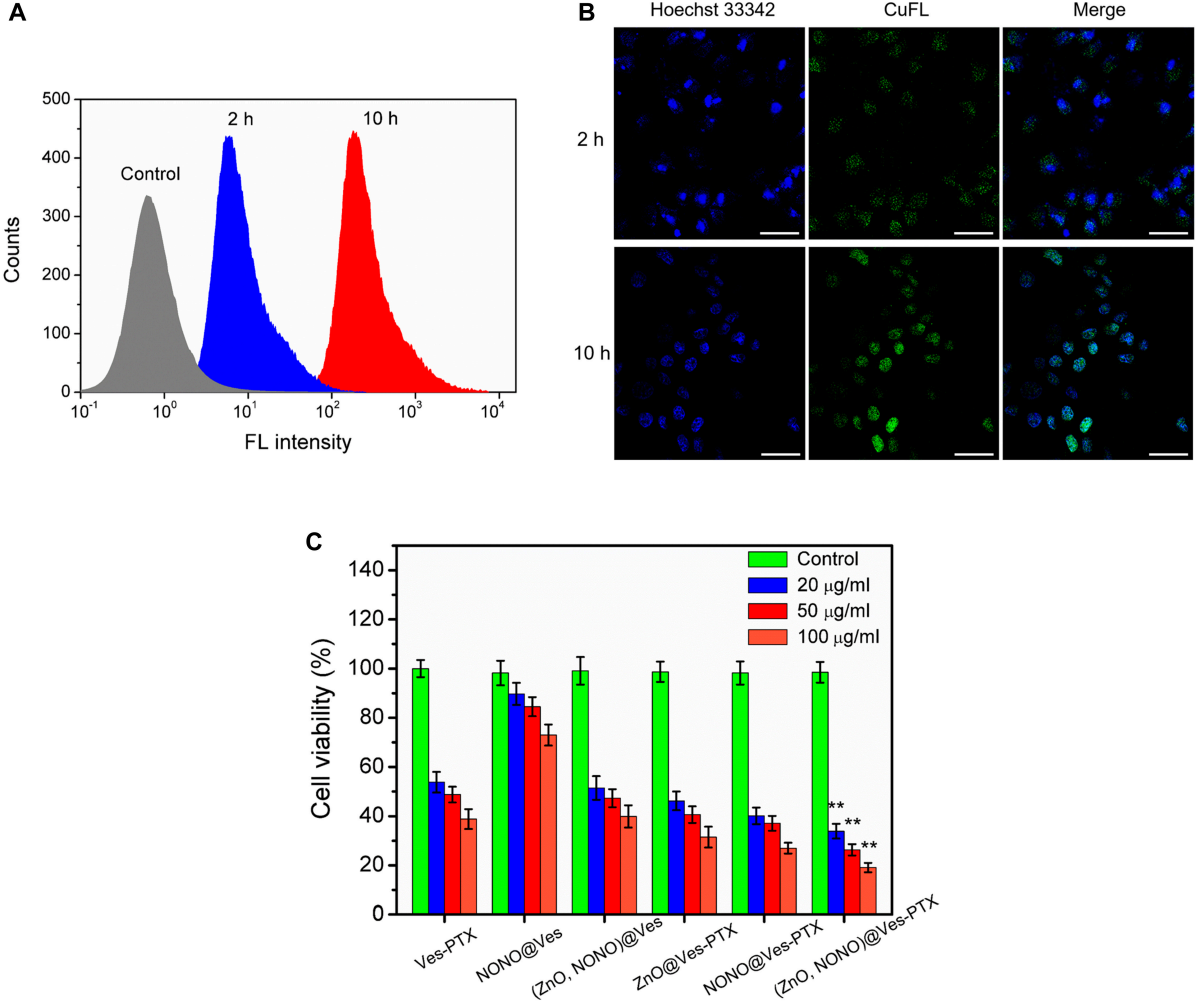
(A) Flow cytometric profiles of A549 cells incubated with (ZnO,NONO)@Ves-PTX for 2 and 10 h. (B) Fluorescence images of A549 cells incubated with (ZnO,NONO)@Ves-PTX for 2 and 10 h at 37 °C. Scale bars, 50 μm. (C) Viability of A549 cells incubated with various drug formulations at different concentrations. Untreated cells were taken as a control. The data are presented as average ± SD (*n* = 5). Statistical significance: **P < 0.01.

To evidence the cell uptake and intracellular NO release of (ZnO,NONO)@Ves-PTX, in vitro cell assay was performed for A549 cells. The A549 cells were first incubated with the NO fluorescent probe, CuFL, to internalize it. Then, the nuclei of the cells were stained with Hoechst 33342. Afterward, the stained cells were incubated with (ZnO,NONO)@Ves-PTX at 37 °C. The fluorescence from the cells after incubation for 2 and 10 h was measured by flow cytometry. As shown in Fig. 7A, the fluorescence emission intensity of the cells from the CuFL encountering NO at 2 h was higher than that of the control, indicating that (ZnO,NONO)@Ves-PTX was internalized by the tumor cells, and NO was released in the cells. The fluorescence intensity became even higher at 10 h, revealing the intracellular release of more NO. The stained A549 cells incubated with (ZnO,NONO)@Ves-PTX were also observed by confocal laser scanning microscopy (CLSM). As displayed in Fig. 7B, the nuclei of the cells emitted blue fluorescence (Hoechst 33342). The green emission from the CuFL in the cells incubated for 10 h was much stronger than that in the cells incubated for 2 h, in accordance with the flow cytometry results. The in vitro cell assay demonstrated the cell uptake of (ZnO,NONO)@Ves-PTX and intracellular NO release from it.

To investigate the inhibitory effect of (ZnO,NONO)@Ves-PTX on lung cancer cells, A549 cells were incubated with various drug formulations at 20, 50, and 100 μg/ml for 24 h, and the cell viability was evaluated by MTT assay. Untreated cells were used as a control for each formulation. As shown in Fig. 7C, the cell viability of the groups was obviously different. Compared with other formulations, (ZnO,NONO)@Ves-PTX had more significant inhibitory effect on the A549 cells. The cell viability of the group treated with NONO@Ves was relatively high, suggesting that the direct anticancer effect of NO was weaker than chemotherapeutic agents. For the same group, the cell viability decreased with the increase of formulation concentration, as the anticancer effect became more remarkable when the concentration increased to higher levels.

### In vivo antitumor study

**Fig. 8.**
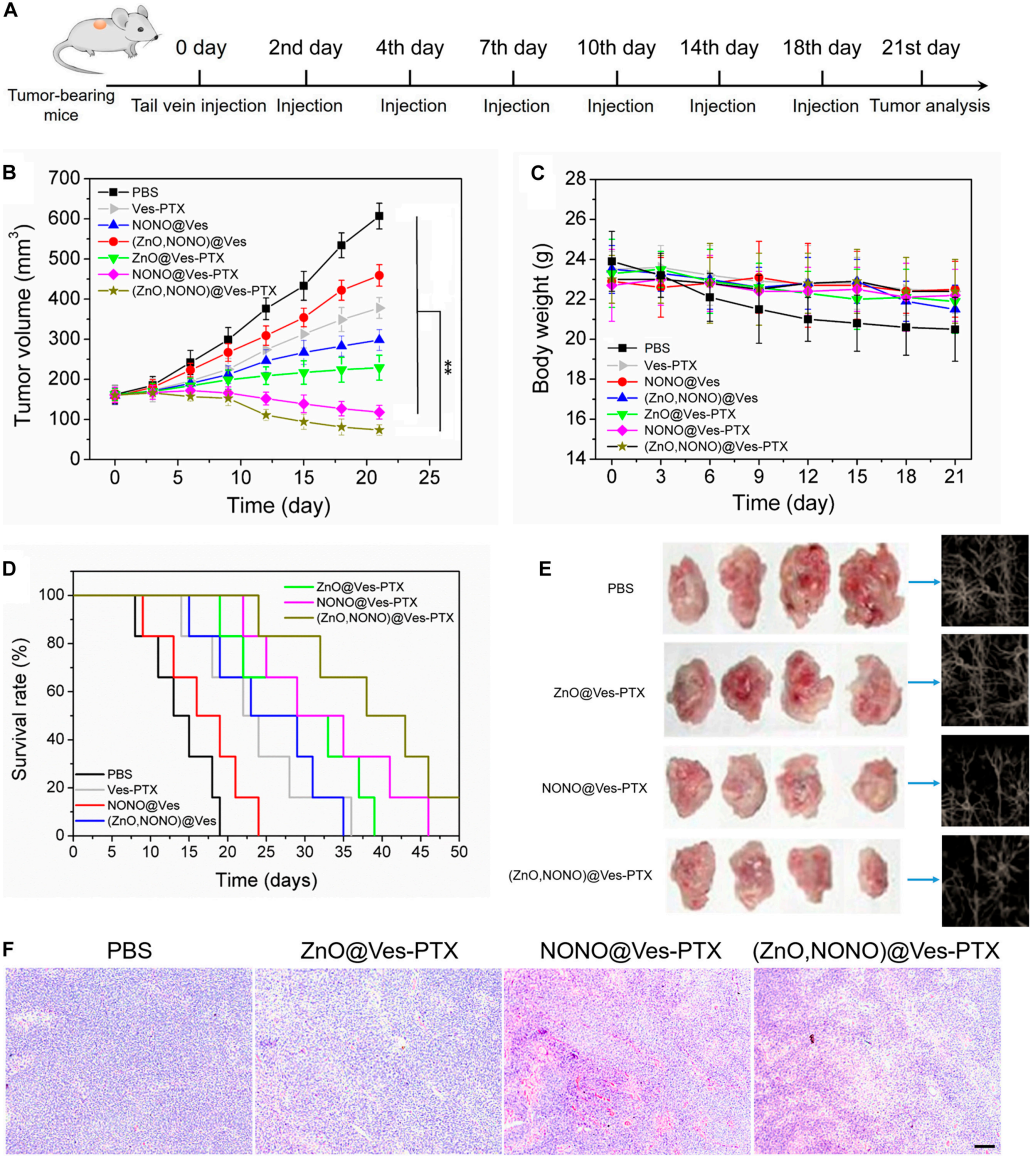
(A) Schedule of delivery strategy of the drug formulations. (B) Volume changes of mice bearing A549 tumors treated with tail vein injection of different formulations. The data are presented as average ± SD (*n* = 5). Statistical significance: **P < 0.01. (C) Body weight changes of the mice after treatment with different formulations. (D) Changes of survival rate with time for the mouse groups treated with different formulations. (E) Pictures of the harvested tumors from differently treated mice before tail vein injection and on the 8th, 15th, and 21st day after injection (from left to right), respectively. Vasculature images of the harvested tumors from differently treated mice on the 21st day after injection (denoted by blue arrows). (F) H&E staining on sections of the A549 tumors from the mice on the 21st day after treatments. Scale bar, 100 μm.

To study the antitumor efficacy of (ZnO,NONO)@Ves-PTX for lung cancer, in vivo animal experiments were performed. Different drug formulations were intravenously injected into BALB/c mice bearing an inoculated A549 tumor as per the strategy depicted in Fig. 8A. The PBS buffer (pH 7.4) was taken as a control. The volume of tumors and the weight of the treated mice were monitored over a 21-d period. As shown in Fig. 8B, the average tumor volume of the control group increased dramatically with time. In contrast, the tumor growth of the treated mice was slower. It was found that delivery of multiple agents exhibited higher antitumor activity than delivery of a single agent, because the agents had synergistic effects. These results agreed with those of the in vitro anticancer experiments, except that NONO@Ves displayed higher anticancer efficacy in vivo than in vitro. In the case of in vitro cell experiments, NONO@Ves could not play the role of vasculature normalization. For in vivo experiments, NONO@Ves released NO in TME, which normalized the tumor vessels. After entering the tumor cells, the released NO inhibited the cell growth. The dual function of NO made NONO@Ves more efficient in tumor therapy. The weight changes of mice after treatment with different formulations are shown in Fig. 8B. The weight of mice in the control group decreased with time. For the treated groups, in comparison, the weight of mice did not decrease obviously within 21 d, revealing that the vesicle-based delivery systems did not exert adverse effects on body.

The mortality of differently treated mice was monitored during a period of 50 d. As shown in Fig. 8D, the mice in the control group all died within 19 d. For those treated with the drug formulations, the survival rate increased to different degrees at the same time points. Among those, the mice treated with (ZnO,NONO)@Ves-PTX survived for the longest time. Sixteen percent of the mice were still alive at the 50th day, indicating that (ZnO,NONO)@Ves-PTX extended the lifetime of the lung tumor-bearing mice.

The tumors harvested from the differently treated mice at certain time points are shown in Fig. 8E. For the mice treated with the PBS buffer, the tumor grew fast and became more irregular with time. Obviously, the drug formulations inhibited the tumor growth. The tumor in a mouse treated with (ZnO,NONO)@Ves-PTX decreased in size with time, indicating the great inhibitory effect of (ZnO,NONO)@Ves-PTX on lung tumors. The vasculature inside the harvested tumors on the 21st day after injection was imaged after the tumor tissue was optically cleared. As shown in Fig. 8E, the tumor vasculature network of the groups treated with NONO@Ves-PTX and (ZnO,NONO)@Ves-PTX had fewer vessel tubes and junctions than those treated with PBS and ZnO@Ves-PTX, convincing the vasculature normalization by the released NO in tumors.

Moreover, H&E staining was performed on sections of the harvested tumors for histopathology analysis. As shown in Fig. 8F, the tumor treated with the PBS buffer had cells with normal morphologies. In contrast, the tumors treated with ZnO@Ves-PTX and NONO@Ves-PTX had abnormal and condensed cells. In the case of mice treated with (ZnO,NONO)@Ves-PTX, the tumor showed more severe condensation and nuclear fragmentation of cells. These results confirmed the inhibitory effect of (ZnO,NONO)@Ves-PTX on tumor growth.

### Safety evaluation

**Fig. 9.**
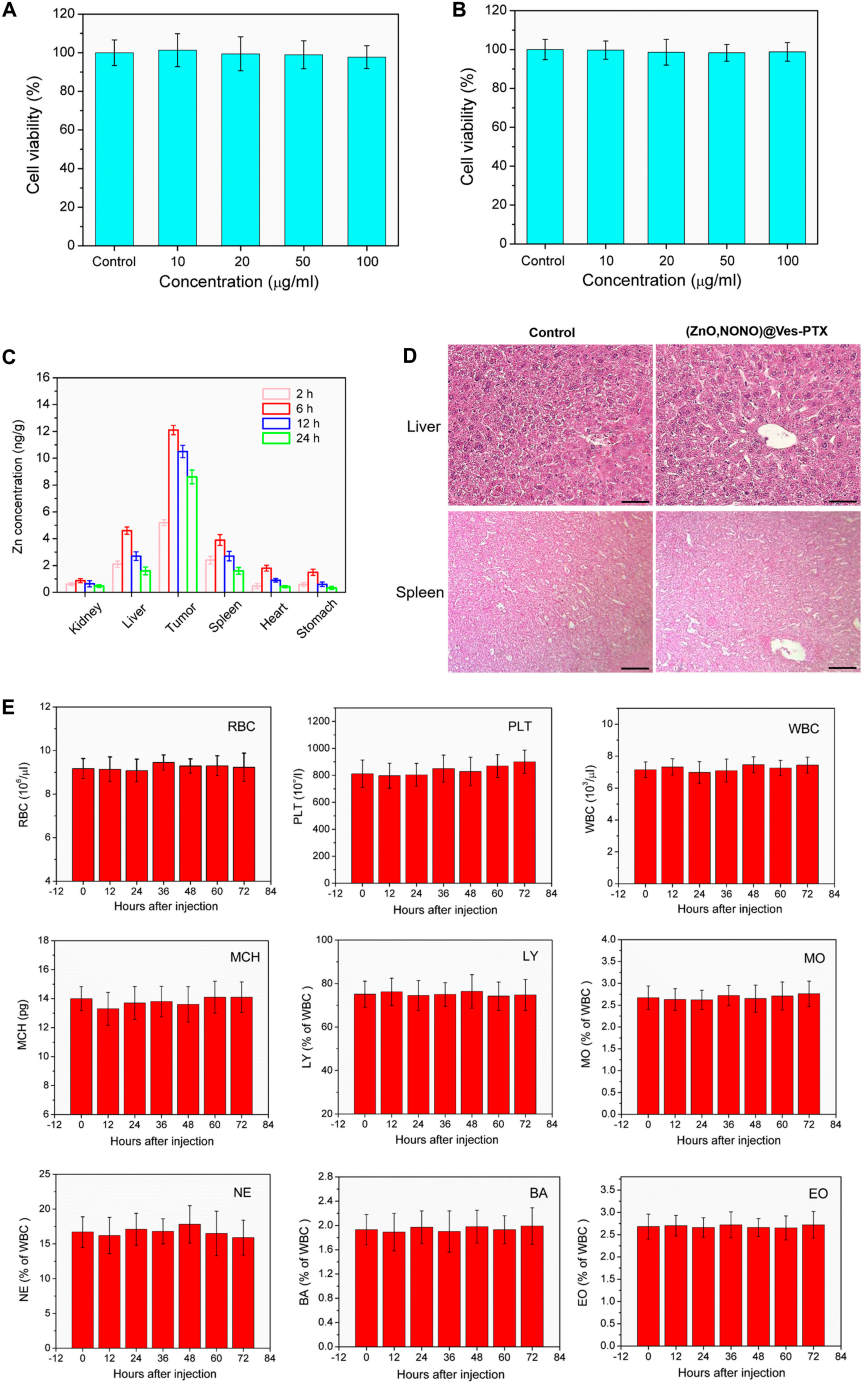
Viability of HUVECs (A) and 3T3 cells (B) incubated with (ZnO,NONO)@Ves-PTX of different concentrations for 24 h. The data are presented as average ± SD (*n* = 4). The data are presented as average ± SD (*n* = 4). (C) Distribution of Zn element in tumor and 5 major organs of the mice sacrificed at different time points after the administration of (ZnO,NONO)@Ves-PTX. The data are presented as average ± SD (*n* = 5). (D) H&E-stained tissue sections of liver and spleen from the mice on the 21st day after treating with PBS buffer (control) and (ZnO,NONO)@Ves-PTX. Scale bar, 100 μm. (E) Blood analysis results of the A549 tumor-bearing mice at different time points after treatment with (ZnO,NONO)@Ves-PTX. The data are presented as average ± SD (*n* = 4). RBC, red blood cells; PLT, platelets; WBC, white blood cells; MCH, mean corpuscular hemoglobin; LY, lymphocytes; MO, monocytes; NE, neutrophils; BA, basophilic cells; EO, eosinophils.

The toxicity of (ZnO,NONO)@Ves-PTX to normal cells was examined by performing MTT assay for HUVECs and 3T3 cells. As shown in Fig. 9A and B, the cell viability of neither HUVECs nor 3T3 cells was affected by the incubation with (ZnO,NONO)@Ves-PTX of different concentrations, suggesting its low cytotoxicity to normal cells.

To further assess the body safety of (ZnO,NONO)@Ves-PTX, the Zn distribution in the tumor-bearing mice at 2, 6, 12, and 24 h after tail vein injection was obtained by ICP-MS. As shown in Fig. 9C, the Ze concentration in the tumor was appreciably higher than those in the organs, as (ZnO,NONO)@Ves-PTX tended to accumulate in the tumor. Among the organs, higher Zn content was found in liver and spleen, revealing that (ZnO,NONO)@Ves-PTX had stronger affinity for the 2 organs. After 6 h, the Zn concentration decreased with time, since (ZnO,NONO)@Ves-PTX was gradually cleared by metabolism. It should be noted that the Zn concentration in the tumor decreased more slowly than in the organs, which means a longer retention of (ZnO,NONO)@Ves-PTX in the tumor. In consideration of relatively high contents of (ZnO,NONO)@Ves-PTX in liver and spleen, the histological sections of the 2 organs at 24 h after injection were analyzed. As can be found in Fig. 9D, the liver and spleen from the mice treated with (ZnO,NONO)@Ves-PTX did not show obvious hyperplasia, inflammation, or necrosis, convincing the body safety of (ZnO,NONO)@Ves-PTX.

Blood analysis was conducted to examine the physiological safety of (ZnO,NONO)@Ves-PTX. After tail vein injection, whole blood was sampled from the treated mice at preset time points during 72 h. Main blood biochemical indices were obtained by blood analysis, as shown in Fig. 9E. All the biochemical indices lay within the normal range at each time point. The normal indices of red blood cells, white blood cells, etc., indicated that the physiological conditions of the treated mice were not influenced by (ZnO,NONO)@Ves-PTX. Lymphocytes, monocytes, basophilic cells, etc., signified no inflammation caused by the treatment. The results of blood biochemical analysis further confirmed the biosafety of (ZnO,NONO)@Ves-PTX.

## Discussion/Conclusion

A nanosystem, (ZnO,NONO)@Ves-PTX, was designed, prepared, and characterized. The DLS results suggested the nanoscale and high colloidal stability of (ZnO,NONO)@Ves-PTX in physiological environment. The GSH-responsive decomposition of (ZnO,NONO)@Ves-PTX, which is imperative for intracellular drug release, was also convinced by DLS.

The on-demand and noninterfering release of NO, PTX, and Zn^2+^ ions, which was verified by drug releasing experiments, demonstrated the ability of (ZnO,NONO)@Ves-PTX to deliver therapeutic agents under intratumoral conditions. The PTX and Zn^2+^ ions are able to kill lung cancer cells synergistically. The released NO plays the roles of normalizing vessels and inhibiting tumor cells. Low-dose NO is capable of normalizing tumor vessels [28]. As (ZnO,NONO)@Ves-PTX is delivered to a tumor, the pH of TME (around 6.5) triggers NO release. The dose of NO will act on tumor vessels and regulate the TME to be more favorable for chemotherapy. Once (ZnO,NONO)@Ves-PTX enters the tumor cells, the intracellular pH (around 5) gives rise to NO release of a larger dose, which will suppress the tumor growth synergistically with other therapeutic agents [42].

In vitro experiments indicated that (ZnO,NONO)@Ves-PTX inhibited angiogenesis by suppressing the expression of tumor angiogenesis factors and migration of HUVECs. In vitro cell assay suggested that (ZnO,NONO)@Ves-PTX could be easily internalized by tumor cells. (ZnO,NONO)@Ves-PTX greatly reduced the viability of tumor cells, but had very low toxicity to normal cells. In vivo experiments using BALB/c mice as the model animals demonstrated remarkable lung tumor inhibitory ability of (ZnO,NONO)@Ves-PTX, originating from the synergistic anticancer effect and vessel normalization. It should be noted that the regimen of 7 injections over 21 d was chosen based on pharmacokinetics showing that such multiple administrations can enhance therapeutic outcomes by maintaining effective drug levels in the tumor over time.

The development of the (ZnO,NONO)@Ves-PTX system represents a great step forward in the field of lung cancer therapy, particularly by integrating the principles of vasculature normalization with chemotherapy. This approach not only enhances drug delivery to the tumor site but also leverages the synergistic effects of NO, ZnO NPs, and PTX to improve therapeutic outcomes.

While our current study focused on lung cancer, the versatility of the (ZnO,NONO)@Ves-PTX system suggests potential applicability to other cancer types. Future research should explore the efficacy of this nanosystem in treating different cancers, including in situ tumor models and metastatic lung cancer models, which more closely mimic the clinical scenarios faced by patients. Also, given the multifunctional nature of the (ZnO,NONO)@Ves-PTX system, future research will explore its integration with other therapeutic modalities, such as immunotherapy or photodynamic therapy. The combination of these approaches could potentially enhance the overall antitumor effect, leading to more robust and durable responses in cancer treatment.

In conclusion, (ZnO,NONO)@Ves-PTX has been developed to remarkably enhance lung cancer chemotherapy. This innovative approach leverages the unique properties of block copolymer vesicles to encapsulate and co-deliver multiple therapeutic agents within a single carrier. The (ZnO,NONO)@Ves-PTX system is designed to release NO in response to the acidic conditions characteristic of the TME and intracellular environments, facilitated by the encapsulated DETA NONOate. The localized release of NO in the TME not only normalizes tumor vasculature but also enhances the permeability and retention of therapeutic agents, thereby optimizing their delivery and distribution.

In conclusion, the decomposition of (ZnO,NONO)@Ves-PTX in response to intracellular GSH levels frees PTX and triggers the release of Zn^2+^ ions from the ZnO NPs. This multi-faceted approach results in a synergistic inhibition of lung tumor growth, with NO-mediated vasculature normalization further potentiating the therapeutic efficacy of the delivered agents. The strategic release of Zn^2+^ ions and NO creates a hostile environment for tumor cells while sparing normal cells, thus minimizing off-target effects. The integration of vasculature normalization with chemotherapy addresses a critical challenge in cancer treatment-efficient drug delivery within TME. The (ZnO,NONO)@Ves-PTX system exemplifies a paradigm shift in chemotherapeutic strategies. The demonstrated high antitumor efficacy, coupled with low systemic toxicity and minimal side effects, positions (ZnO,NONO)@Ves-PTX as a promising candidate for clinical translation.

## Data Availability

Data will be made available on request.
