# Supplementary material for: A Polymeric Vesicle System for Combined Lung Cancer Therapy through Chemotherapy and Vasculature Normalization
Source: Biomater Res. 2024 Nov 27;28:0117. doi: 10.34133/bmr.0117 (PMC11599482; doi:10.34133/bmr.0117)
Supplement: Supplementary 1 — Figs. S1 to S3 [file bmr.0117.f1.docx]

**A Polymeric Vesicle System for Combined Lung Cancer Therapy through Chemotherapy and Vasculature Normalization**

Ding Wang^a#^, Cheng-Jie Qiu^b#^, Yaoqing Chu^a#^, Anzhuo Zhang^a^, Ran Huang^c,d*^, Si-Jian Pan^b*^, Lianjiang Tan^a,b*^

^a^ School of Materials Science and Engineering, Shanghai Institute of Technology, Shanghai 201418, China.

^b^ Department of Neurosurgery, Ruijin Hospital, Shanghai Jiao Tong University School of Medicine, Shanghai 200025, China.

^c^ Academy for Engineering and Applied Technology; Yiwu Research Institute; Zhuhai Fudan Innovation Institute, Fudan University, Shanghai 200433, China.

^d^ Center for Innovation and Entrepreneurship, Taizhou Institute of Zhejiang University, Taizhou, Zhejiang 318000, China.

^#^ These authors contributed equally to the paper.

^*^Corresponding author. Email: [tanlianjiang@126.com](mailto:tanlianjiang@126.com); [psj11629@rjh.com.cn](mailto:psj11629@rjh.com.cn); [huangran@fudan.edu.cn](mailto:huangran@fudan.edu.cn).

**Supporting information**


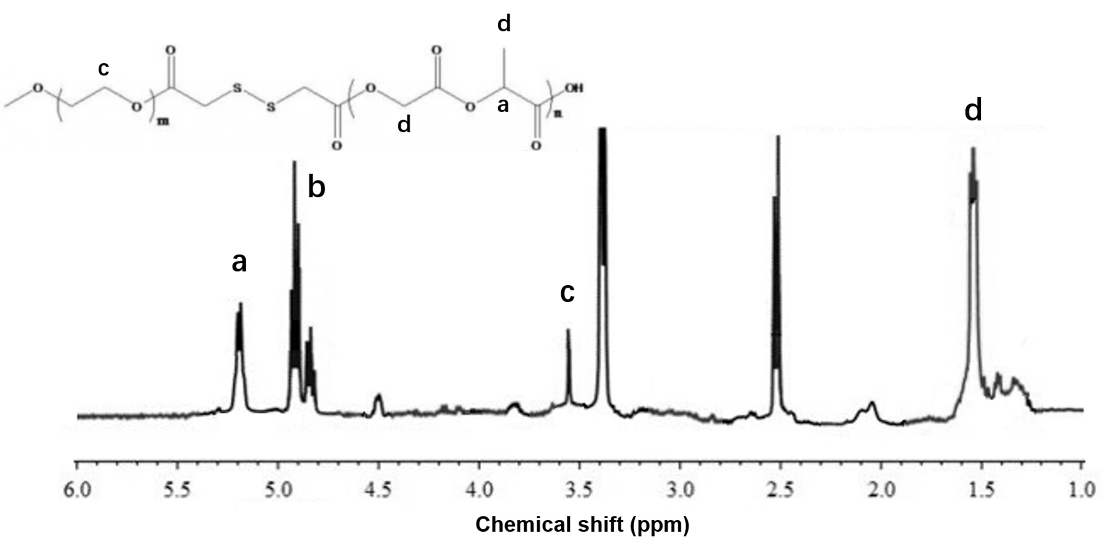


**Fig. S1.** ^1^H NMR spectra of the copolymer PEG_2000_-SS-PLGA_1000_.





**Fig. S2.** Zeta potential of (ZnO,NONO)@Ves-PTX in water.





**Fig. S3.** Tube formation of HUVECs as a function of NO concentration.
